# Celiac Disease–Specific TG2-Targeted Autoantibodies Inhibit Angiogenesis *Ex Vivo* and *In Vivo* in Mice by Interfering with Endothelial Cell Dynamics

**DOI:** 10.1371/journal.pone.0065887

**Published:** 2013-06-18

**Authors:** Suvi Kalliokoski, Ana-Marija Sulic, Ilma R. Korponay-Szabó, Zsuzsa Szondy, Rafael Frias, Mileidys Alea Perez, Stefania Martucciello, Anne Roivainen, Lauri J. Pelliniemi, Carla Esposito, Martin Griffin, Daniele Sblattero, Markku Mäki, Katri Kaukinen, Katri Lindfors, Sergio Caja

**Affiliations:** 1 Tampere Center for Child Health Research, University of Tampere and Tampere University Hospital, Tampere, Finland; 2 Celiac Disease Center, Heim Pál Children's Hospital, Budapest and Department of Paediatrics, Medical and Health Science Center, University of Debrecen, Debrecen, Hungary; 3 Department of Biochemistry and Molecular Biology, Medical and Health Science Center, University of Debrecen, Debrecen, Hungary; 4 Central Animal Laboratory, University of Turku, Turku, Finland; 5 School of Life and Health Sciences, Aston University, Birmingham, United Kingdom; 6 Department of Chemistry and Biology, University of Salerno, Salerno, Italy; 7 Turku PET Centre, Turku University Hospital and University of Turku and Turku Center for Disease Modeling, University of Turku, Turku, Finland; 8 Laboratory of Electron Microscopy, University of Turku, Turku, Finland; 9 Department of Health Sciences and IRCAD, University of Eastern Piedmont, Novara, Italy; 10 School of Medicine, University of Tampere and Department of Gastroenterology and Alimentary Tract Surgery, Tampere University Hospital, Tampere, and Department of Medicine, Seinäjoki Central Hospital, Seinäjoki, Finland; University of Texas MD Anderson Cancer Center, United States of America

## Abstract

A characteristic feature of celiac disease is the presence of circulating autoantibodies targeted against transglutaminase 2 (TG2), reputed to have a function in angiogenesis. In this study we investigated whether TG2-specific autoantibodies derived from celiac patients inhibit angiogenesis in both *ex vivo* and *in vivo* models and sought to clarify the mechanism behind this phenomenon. We used the *ex vivo* murine aorta-ring and the *in vivo* mouse matrigel-plug assays to address aforementioned issues. We found angiogenesis to be impaired as a result of celiac disease antibody supplementation in both systems. Our results also showed the dynamics of endothelial cells was affected in the presence of celiac antibodies. In the *in vivo* angiogenesis assays, the vessels formed were able to transport blood despite impairment of functionality after treatment with celiac autoantibodies, as revealed by positron emission tomography. We conclude that celiac autoantibodies inhibit angiogenesis *ex vivo* and *in vivo* and impair vascular functionality. Our data suggest that the anti-angiogenic mechanism of the celiac disease-specific autoantibodies involves extracellular TG2 and inhibited endothelial cell mobility.

## Introduction

Angiogenesis, the formation of blood vessels, has emerged as an essential phenomenon involved in various disorders. Also intestine-related diseases, such as inflammatory bowel disease, ascites and peritoneal adhesions, are characterized or contributed by dysregulated blood vessel growth or formation [1]. In inflammatory bowel disease, for instance, it has been demonstrated that increased vascularization is present in the inflamed colonic mucosa of the patients and the expression of several angiogenic factors is markedly increased [2], [3]. Similarly, untreated celiac disease patients have been reported to evince abnormalities in their small-intestinal mucosal vasculature [4], [5]. In addition to these vascular aberrations, untreated celiac patients have disease-specific circulating autoantibodies targeted against transglutaminase 2 (TG2) in their sera and as deposits in their small-intestinal mucosa. In the mucosa autoantibodies are bound to TG2 below the epithelium on the basement membrane and interestingly also around blood vessels [6], [7].

The target of the celiac autoantibodies, TG2, is a ubiquitously expressed enzyme involved in a wide range of cellular processes including angiogenesis. TG2, expressed highly by endothelial cells, contributes to angiogenesis by cross-linking a variety of extracellular matrix (ECM) proteins through the formation of Ca^2+^-dependent covalent linkages [8], [9]. Celiac disease-specific TG2-targeted autoantibodies have been proposed to disturb endothelial cell biology *in vitro* [10], [11], but information about their capability to interfere with vessel formation and function in more complex *ex vivo* and *in vivo* systems is not available. This study was designed specifically to address the question what kind of effects the celiac disease-specific autoantibodies have on vascular formation and functionality *ex vivo* and *in vivo* and to discover the mechanism behind.

## Materials and Methods

### Ethics statement

The protocol for mouse studies was approved by the Finnish and Hungarian authorities, the Turku Central Animal Laboratory (University of Turku, Finland) and the Debrecen University animal facility (Debrecen, Hungary). The study protocol for using human serum samples was approved by the Ethics Committee of Tampere University Hospital, Tampere, Finland, and written informed consent was received from all subjects.

### Animals

For *ex vivo* and *in vivo* studies, 4–6-week-old female Balb/c mice (Harlan Laboratories Inc. Horst, the Netherlands) or C57BL/6 wild type or TG2 knockout mice [12], were housed at 22°C in a 12-hour light/dark cycle with water and food freely available. The animals were cared for and used in accordance with the regulations in Finland, Hungary and the European Union (86/609/EC).

### Purification of serum IgA and production of monoclonal antibodies

Serum samples from three biopsy-proven celiac disease patients on a gluten-containing diet and positive for both anti-TG2 (>100 U/ml; Celikey, Phadia GmbH, Freiburg, Germany) and endomysial antibodies (1:>2,000) were employed in the study. As controls we used serum samples from three non-celiac controls, which all were negative for the above-mentioned antibodies. Total IgA fractions from serum samples were purified as previously described [10], using cyanogen bromide-activated Sepharose 4B (Pharmacia Upjohn, Uppsala, Sweden) coupled with 7 mg/ml rabbit anti-human IgA antibodies (Sigma Aldrich, St Louis, MO, USA). Thereafter, the IgA samples were lyophilized and resolubilized in Hank's balanced salt solution to a final concentration of 100 µg/ml. Purified antibodies were used in the experiments at a concentration of 1 µg/ml.

The following IgG-class recombinant monoclonal autoantibodies prepared from celiac patients were used: celiac patients' anti-TG2 specific monoclonal antibodies targeting the major celiac epitope; clone 4.1 (CD Mab) and irrelevant control antibodies (clones 5.1 and 6.2, non-CD Mab) targeted against Escherichia coli proteins M5 and M6 [13], [14]. Recombinant technology was essentially applied in Chinese hamster ovary cells to produce the monoclonal antibodies as previously described [15], which were used in the experiments at a concentration of 1 µg/ml.

### *Ex vivo* aorta ring and *in vivo* matrigel plug angiogenesis assays

Mouse aortas were cut into 0.5 mm-thick rings and embedded in matrigel (BD Biosciences, Bedford, MA, USA) containing CD Mabs or their respective controls, non-CD Mabs. The aorta rings were cultured in EGM-2 plus endothelial cell growth factors provided in the EGM-2 Bulletkit (Clonetics, San Diego, CA, USA) for ten days. Thereafter, images of endothelial sprouts and interconnected capillary tubes were randomly taken using a Zeiss inverted microscope and Axiovision 3.0 program (Carl Zeiss Vision GmbH, München-Hallbergmoos, Germany). The pictures were analyzed using ImageJ software (http://rsb.info.nih.gov/ij) [16].

*In vivo* mouse angiogenesis assays were performed using matrigel (BD Biosciences) containing 10 µg/kg of erythropoietin (EPO; Sigma Aldrich, St. Louis, MO, USA), which was injected subcutaneously into the backs of mice. This system is based on the EPO-induced self-production of vessels by cells migrating into the matrigel plugs from the host [17]–[20]. CD Mabs or their respective controls were mixed with matrigel and injected to recipient mice. After eight days, some of the mice were examined by positron emission tomography (PET). Finally, the matrigel plugs were removed from all animals, snap-frozen and stored at −80°C for further analysis.

### *In vitro* angiogenesis assays

*In vitro* assays were performed using human umbilical vein endothelial cells (HUVECs) purchased from Lonza (Cambrex Bio Science, Walkersville, MD, USA). HUVECS were cultured at 37°C and 5% CO^2^ in endothelial growth medium-1 (EGM-I; Clonetics). EGM-I consists of endothelial cell basal medium (EBM-I; Clonetics) and endothelial cell growth factors provided in the EGM-I Bulletkit (Clonetics).

HUVECs (2.5×10^5^cells/well) were mixed with matrigel (BD Biosciences; diluted 1∶3 in EGM-1) and celiac patient-derived IgA (CD IgA) or CD Mab or their respective controls (non-CD IgA and non-CD Mab). In a subset of experiments we used an active site-directed irreversible extracellular TG2 inhibitor R281 at a concentration of 200 µM [21]. In this case, the inhibitor was administered one hour prior to addition of antibodies. After 48 hours images of different fields were randomly taken using a Zeiss inverted microscope and Axiovision 3.0 program (Carl Zeiss Vision GmbH, München-Hallbergmoos, Germany). Length, branch, area and number of endothelial tubules were measured using ImageJ analysis software.

In addition, HUVECs embedded in matrigel as described above were cultured for ten days in Cell-IQ (Chip-man Technologies LTD, Tampere, Finland). Briefly, the system consists of a cell incubator containing an integrated system designed to take and analyze images. During the assays images were taken every five minutes. The Cell IQ software was used for further enumeration of apoptotic and immobile cells and video editing. For the analysis of cell movements cells were tracked using MTrack tool in ImageJ software.

### *In vivo* vascular functionality assay

Balb/c mice, injected subcutaneously with matrigel plugs as described above, received an intravenous injection of Hoechst 33342 (Hoechst; Invitrogen, Carlsbad, CA, USA) five minutes before euthanasia. Thereafter, the plugs were snap-frozen after excision and stored at −80°C until further analysis.

### Positron emission tomography

In this experiment one mouse received three matrigel implants, each treated with PBS (basal group), non-CD Mab or CD Mab and injected subcutaneously into separate limbs. Mice were anesthetised with isoflurane and intravenously injected with 2-[^18^F]-fluoro-2-deoxy-D-glucose ([^18^F]FDG). PET imaging for 20 minutes was performed using an Inveon Multimodality scanner (Siemens Medical Solutions, Knoxville, TN) at 60 minutes post injection and reconstructed with the ordered-subsets expectation maximization 2D algorithm (OSEM2D). After PET imaging the animals were euthanised and the matrigel plugs excised, weighed and measured for radioactivity using a gamma counter (Triathler 3″, Hidex, Turku, Finland), which was cross-calibrated with a dose calibrator (VDC-202, Veenstra Instruments, Joure, the Netherlands). Quantitative analysis was performed by drawing regions of interest in the matrigel plug areas (Vinci software, version 2.54; Max Planck Institute for Neurological Research, Cologne, Germany). The average radioactivity concentration (kBq/ml) in the regions of interest was used to verify *in vivo* results.

### Electron microscopy

Aortas collected from the mice and treated as described above were prepared for electron-microscopy analysis. Briefly, the specimens were cut to 1 mm thickness, fixed in 5% glutaraldehyde (Electron Microscopy Sciences, Fort Washington, PA, USA) in 0.16 M s-collidin buffer (pH 7.4) and post-fixed with potassium ferrocyanide-osmiun tetroxide as previously described [22]. Samples were embedded in epoxy resin (Glycidether 100, Merck, Darmstadt, Germany) and cut into thin sections (70 nm) [22]. Sections were stained with 5% uranyl acetate and 5% lead citrate in an Ultrostainer (Leica, Wien, Austria) and examined under a JEM-100SX transmission electron microscope (JEOL, Tokyo, Japan).

### Immunofluorescence studies

Frozen sections from mouse matrigel plugs were fixed in 4% paraformaldehyde and processed to 5–7-µm-thick sections. The presence of vessels in the matrigel implants was studied by immunofluorescent labeling with an anti-von Willebrand factor (vWF) antibody (1∶200, Dako, Glostrup, Denmark) and counterstained with a secondary antibody labeled with Alexa-488 (1∶1000, Molecular Probes, Eugene, OR, USA) at room temperature. Nuclei were stained with Vectashield mounting medium containing 4′,6-diamidino-2-phenylindole (DAPI; Vector Laboratories, Inc., Burlingame, CA, USA). Images were taken with an Olympus BX60 microscope (Olympus Europa GmbH, Hamburg, Germany) and Cell^D^ imaging software (Olympus Europa GmbH, Hamburg, Germany). Quantitative data were obtained by counting the number and diameter of vWF-positive blood vessels as well as the total number of cells per matrigel area in digitalized images using ImageJ software.

### Demonstration of TG2 in the matrigel

First, 100 µl of matrigel (BD Biosciences) was jellified and mixed with 100 µl of Laemmli-buffer. After ten minutes the samples were sonicated and centrifuged for ten minutes at 12 000 rpm at 4°C. Subsequently, the samples were concentrated with acetone. The protein concentrations were measured by the Bradford method and 20 µg of total protein was loaded on 10% polyacryl amide gels (1.5 M Tris–HCl, pH 8.8; sodium dodecyl sulfate, 12%; acrylamide/bis-acrylamide, 30%; APS, 10%; and TEMED), followed by transfer to a Hybond-P membrane (Amersham Biosciences, Little Chalfont, Bucks, UK). After blocking in 5% milk, the membrane was incubated with mouse monoclonal antibody against TG2, CUB7402 (1∶200, Santa Cruz Biotechnology Inc, Santa Cruz, CA, USA), followed by a secondary rabbit antibody conjugated with horseradish peroxidase. Detection was performed with the ECL PlusWestern Blotting Detection System (ECL, GE Healthcare Biosciences, Pittsburgh, PA, USA).

### Statistical analysis

Statistical comparisons were made using statistical analysis software (PASW Statistics 18, SPSS Inc., Chicago, IL, USA). The data were first tested for homogeneity. When the data fulfilled this criterion and there were three or more groups involved in an experiment, one-way ANOVA analysis was used. A two-way ANOVA within subjects was used to compare the effect of TG2 inhibitor with respect to each treated group. In both cases a Student Newman Keuls (SNK) test was performed as *post hoc* analysis. Non-homogeneous data were compared with Kruskal-Wallis test and further tested by Mann-Whitney U test. The data are presented as mean ± standard error of mean (SEM), a p-value≤0.05 being considered significant.

## Results

### Celiac disease antibodies impair angiogenesis *ex vivo* and *in vivo*

**Figure 1:**
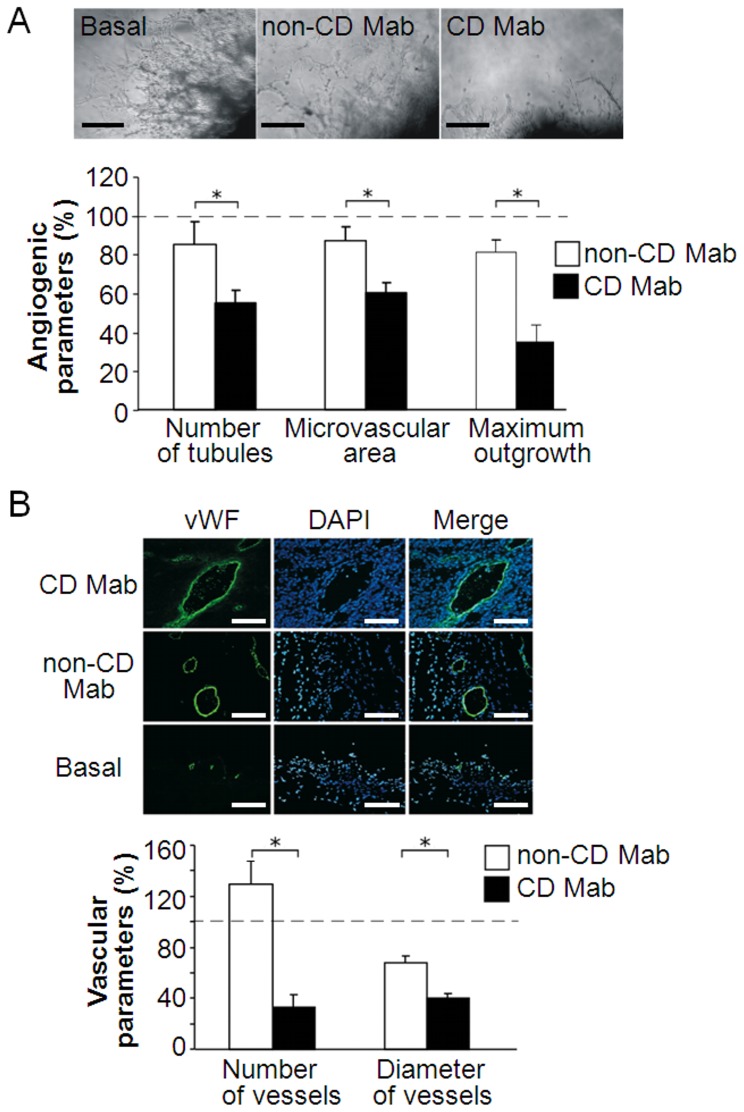
Angiogenesis is impaired *ex vivo* and *in vivo* after treatment with celiac disease patient monoclonal autoantibodies. (A) Representative figures taken from mouse aorta rings cultured inside matrigel for ten days in the presence of monoclonal celiac disease-specific transglutaminase 2 antibody (CD Mab) or its respective control (non-CD Mab). Scale bars represent 100 µm. Bars represent different angiogenic parameters. (n = 4 aortas per group). (B) Representative pictures of mouse matrigel implants treated with CD Mab or non-CD Mab. After eight days the implants were removed and stained for blood vessels with von Willebrand factor (vWF)-antibody (green) and blue color indicates nuclei (DAPI). Scale bar represents 200 µm. Bars show vascular parameters measured from matrigel implants. (n = 8 animals per group). Bars in both charts, A and B, represent the average value as percentage + SEM. All data was normalized to the basal group (dotted line). * represents P≤0.001 statistical difference.

To investigate the effects of celiac disease antibodies on angiogenesis occurring in an assay, which recapitulates all the key steps in the process including matrix degradation, migration, proliferation and reorganization, we performed e*x vivo* aorta ring experiments. Using this system, we observed that the number of the tubules, the microvascular area and the maximum microvessel outgrowth were significantly reduced in the presence of CD Mab as compared to non-CD Mab (P≤0.001; Figure 1A). As definitive tests for angiogenesis require *in vivo* experiments, we next performed mouse matrigel experiments. In this assay our results were parallel to those obtained from aorta ring experiments as both the number and diameter of the vessels inside the matrigel were significantly decreased in the presence of CD Mab (P≤0.001; Figure 1B).

**Figure 2:**
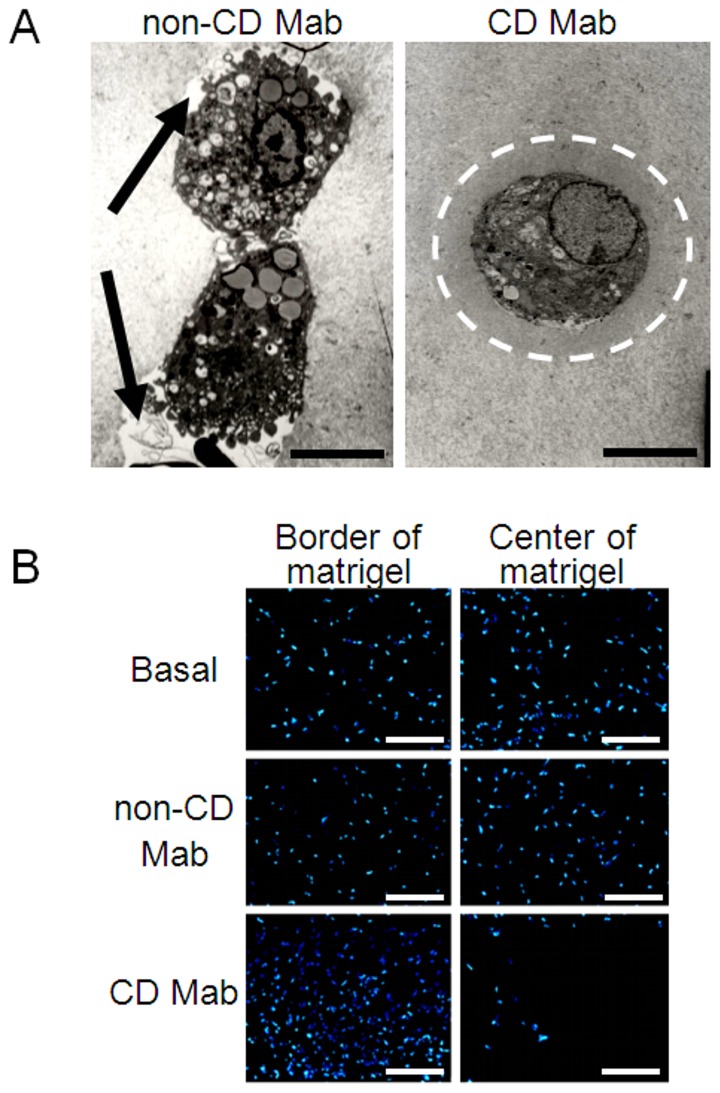
Cellular behavior is altered in the presence of celiac autoantibodies. (A) Transmission electron micrographs of mouse aorta ring experiments in the presence of monoclonal celiac patient-derived transglutaminase 2-targeted antibody (CD Mab) or its respective control (non-CD Mab). Arrows indicate the extracellular matrix (ECM)-free area in the leading edges of the cells treated with non-CD Mab and the white dotted line shows an area of densely organized ECM around a cell treated with CD Mab. Scale bar represents 6 µm. (B) Representative images taken from mouse matrigel implants treated with CD Mab or non-CD Mab. Nuclei are shown as blue (DAPI) from the border and center of the implants. Scale bar represents 300 µm.

To gain further insight into the effects of celiac antibodies, we took transmission electron microscope images from *ex vivo* mouse aorta ring assays, and observed that in control cultures there were pseudopodia in the protruding leading edges of cells and pericellular areas free of ECM (Figure 2A). In contrast, in the presence of celiac patient TG2-targeted autoantibodies cells outgrowing from mouse aortas were round and did not exhibit cellular processes characteristic for the leading edge during migration. Furthermore, the mouse matrigel implants were used to study the localization of cells migrating from the mice to the plug. We found that in the basal group and in the group treated with non-CD Mab, cells had migrated from the animal even to the center of the matrigel and were evenly distributed inside the matrigel implant. In contrast, in the matrigels treated with CD Mab, cells were mainly located in the border of the matrigel and only few were found in the central area (Figure 2B).

### Dynamics of endothelial cell behavior is altered in the presence of celiac disease antibodies

**Figure 3:**
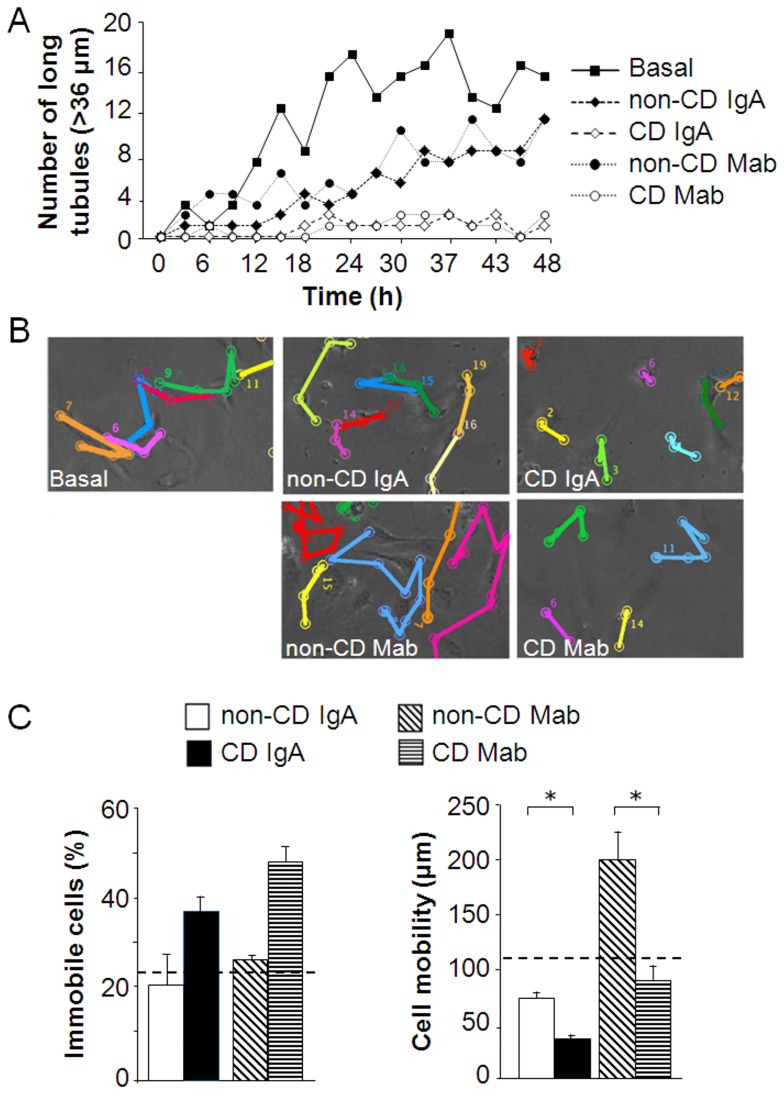
Endothelial cell dynamics in the presence of celiac patient-derived total IgA (CD IgA) or monoclonal antibodies (CD Mab), or their relevant controls (non-CD IgA or non-CD Mab). (A) The number of long tubules (>36 µm) was counted from videos taken from human umbilical vein endothelial cell (HUVEC) cultures during a 48 hour time course in the presence of CD IgA or CD Mab, or their relevant controls non-CD IgA or non-CD Mab. (B) HUVECs were tracked for 48 hours and cellular mobility during that time course is indicated in representative pictures. Each color represents an individual cell. (C) The percentage of immobile cells (left panel) and the cell mobility in micrometers (right panel) were determined in the cell cultures treated with CD IgA or CD Mab or their relevant controls. Bars represent the average value as percentage + SEM. Basal group is indicated as a dotted line. * represents P≤0.001 statistical difference.

The data obtained from aorta ring and mouse matrigel experiments suggested that the anti-angiogenic effects exerted by celiac antibodies could be contributed by defective migration. To study this further we took videos of HUVECs growing inside matrigel. In cultures supplemented with control antibodies, the formation of tubule-like structures was evident after 20 hours of culture. However, in the presence of celiac antibodies (both total CD IgA and CD Mab) the formation of tubule-like structures, especially long ones (>36 µm), was inhibited when compared to controls during the 48 hour time course (Figure 3A, Video S1). Concurrently, the number of branches and tubules after 48 hours of culture were significantly reduced in the cultures treated with IgA and CD Mab compared to controls (Figure S1).

Tracking of cells grown inside matrigel revealed that in the presence of celiac antibodies the number of immobile cells was increased. The cells that did move regardless of the presence of celiac antibodies, moved half of the distance of those cultured in the presence of their relevant control antibodies (P≤0.001). However, cellular mobility showed extensive variability among experimental groups (Figure 3B, C). To exclude the possibility that the anti-angiogenic effects of celiac disease antibodies are due to increased apoptosis, we quantified the number of apoptotic cells and found that their number was not increased in the presence of celiac antibodies (Figure S2).

### Celiac disease TG2-specific antibodies affect *in vivo* vascular functionality

**Figure 4:**
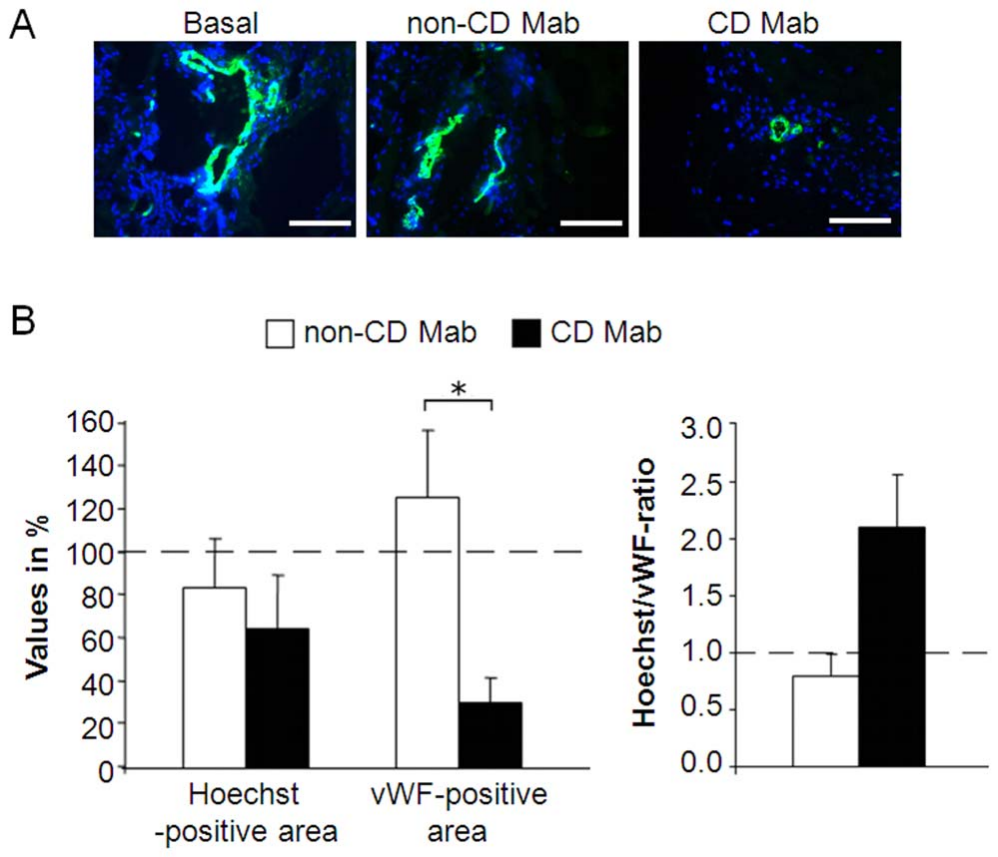
Functionality of vessels in mice in the presence of celiac patient-derived monoclonal autoantibodies (CD Mab) or their relevant controls (non-CD Mab). (A) Representative images of mouse matrigel implants were treated with CD Mab or non-CD Mab. Hoechst 33342 (Hoechst) was injected intravenously into a tail vein five minutes prior to euthanasia. The removed implants were stained for blood vessels with anti-von Willebrand factor (vWF)-antibody (green). Blue color indicates Hoechst-stained nuclei (DAPI) and scale bar represents 200 µm. (B) Vascular functionality parameters were determined from matrigel implants (left panel) and the ratio of Hoechst- and vWF-positive area was calculated (right panel). (n = 4 animals per group). Bars represent the average value as percentage + SEM. All data was normalized to the basal group (dotted line). * represents P≤0.05 statistical difference.

We next studied capillary perfusion in *in vivo* matrigel implants using an intravenous tail vein injection of Hoechst [23]. In all treatment groups the vessels inside the matrigel were functional, since in each case the injected dye was located around blood vessels (Figure 4A). The percentage of the Hoechst-positive area was similar in all groups, whereas the area occupied by vWF-staining was significantly reduced in the group treated with CD Mab (P≤0.05). The ratio between the Hoechst- and vWF-positive areas was 2.6 times higher in the group treated with CD Mab than in the relevant control (Figure 4B).

**Figure 5:**
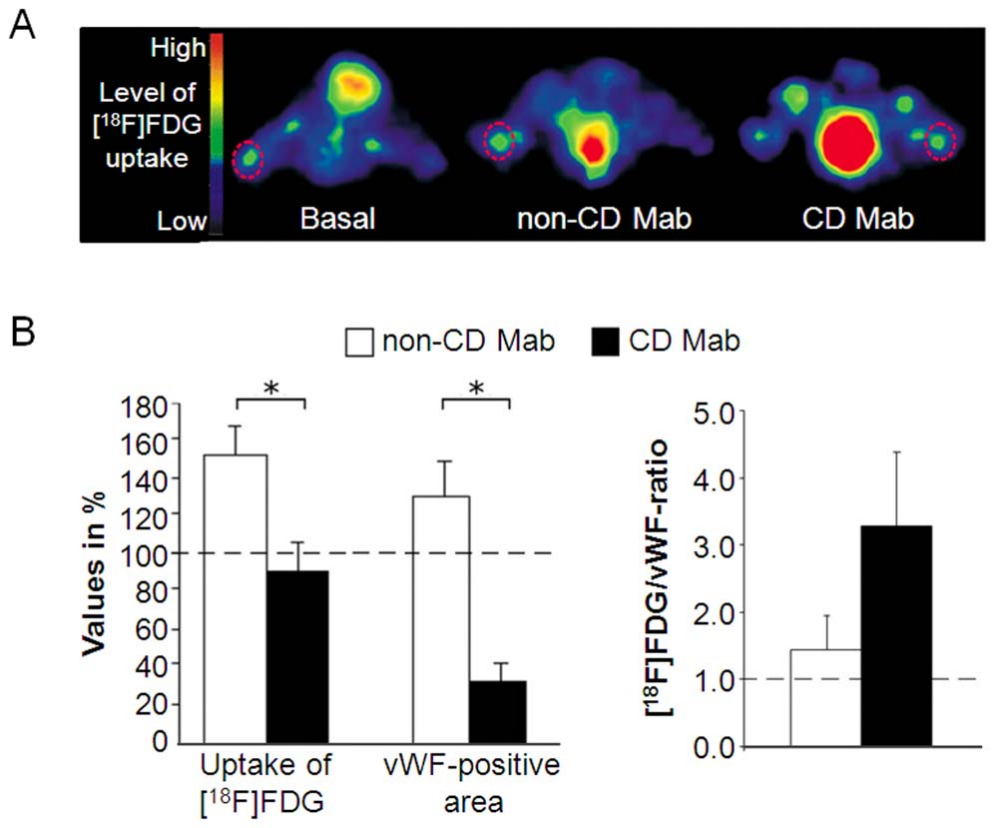
Vessel functionality studied by positron emission tomography (PET). (A) 2-[^18^F]-fluoro-2-deoxy-D-glucose ([^18^F]FDG) PET scans of mice implanted with matrigel (basal) or supplemented with celiac disease-specific monoclonal autoantibodies (CD Mab) or control antibodies (non-CD Mab). Each mouse received three matrigel implants (basal, CD Mab or non-CD Mab), each injected subcutaneously into separate limb as highlighted by circles in the scan. The magnitude of [^18^F]FDG uptake is defined in different colors. (n = 3) (B) The uptake of [^18^F]FDG in the implants was analyzed from the PET images and the area occupied by vessels was determined by von Willebrand factor (vWF)-antibody staining (left panel). The ratio of [^18^F]FDG uptake and vascular area was calculated (right panel). Bars represent average value as percentage + SEM. All data was normalized to the basal group (dotted line). * represents P≤0.05 statistical difference.

The functionality of the vessels formed inside *in vivo* matrigel implants was also studied by PET (Video S2). We found that [^18^F]FDG uptake was decreased in the matrigel treated with CD Mab in comparison with non-CD Mab (P≤0.05) but not with basal group. However, the matrigel implants treated with CD Mab contained significantly fewer vessels than controls (P≤0.05). The ratio of [^18^F]FDG uptake and vessel area was increased more than two fold in CD Mab-treated matrigel implants than in the controls (Figure 5A, B).

### The contribution of extracellular TG2 in the anti-angiogenic response

**Figure 6:**
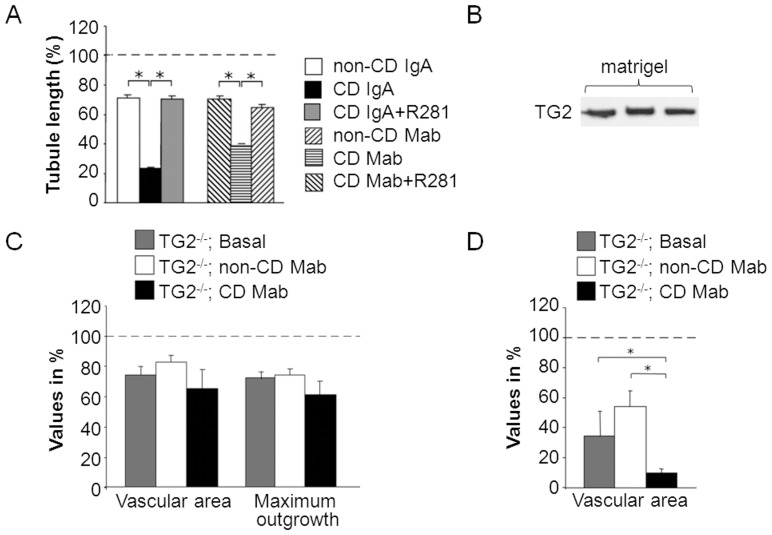
The contribution of extracellular transglutaminase 2 (TG2) in the anti-angiogenic response exerted by celiac antibodies. (A) Endothelial tubule length in three-dimensional human umbilical vein endothelial cell (HUVEC) cultures in the presence of celiac patient-derived total IgA (CD IgA) or monoclonal antibodies (CD Mab), or their relevant controls (non-CD IgA or non-CD Mab) and with/without TG2-inhibitor R281. All data was normalized to the basal group (dotted line). Bars represent the average value as percentage + SEM. * represents P≤0.001 statistical difference (n = 9). (B) Western blot using anti-TG2 antibody CUB7402 showing the presence of TG2 in the matrigel. (C) Mouse aorta rings derived from TG2 knockout mice (TG2^−/−^) were cultured inside TG2-containing matrigel for ten days without supplementation (basal) or in the presence of CD Mab or non-CD Mab. After the culture period the vascular area and the maximum outgrowth were measured (n = 5 aortas per group). (D) *In vivo* angiogenesis assays were performed in TG2^−/−^ mice. Matrigel containing endogenous TG2 was supplemented with CD Mab or non-CD Mab and injected to mice. After eight days the implants were removed and the vascular area determined by anti-von Willebrand factor (vWF)-antibody staining. (n = 8 animals per group). Bars in charts C and D represent the average value as percentage + SEM. All data was normalized to the wild type mouse basal group (dotted line). * represents P≤0.01 statistical difference.

As extra- and intracellular TG2 have been demonstrated to be involved in distinct endothelial cell processes [24], we next studied whether the anti-angiogenic response by celiac disease antibodies is mediated by cellular or extracellular TG2. To this end, we first tested the role of extracellular TG2 by pretreating HUVEC cultures with the cell-impermeable TG2 inhibitor R281 and found that the inhibitor was able to restore the tubule length of CD IgA- or CD Mab-treated cells to control level (P≤0.001; Figure 6A).

To confirm the contribution of extracellular TG2, we performed experiments using TG2 knockout mice. By definition, TG2 knockout mice do not express cellular TG2 [12], whereas Figure 6B clearly demonstrates that matrigel itself, used in the following experiments as extracellular growth environment, contains significant amounts of extracellular TG2. *Ex vivo* aorta ring assay with TG2 knockout mouse tissue showed that in the presence of CD Mab the microvessel and maximum outgrowth areas were decreased slightly but not significantly (Figure 6C). On the other hand, the *in vivo* matrigel plug assays performed in the knockout animals demonstrated that the vascular area in the implants was significantly reduced after CD Mab treatment (P≤0.01; Figure 6D). It is of note that the overall angiogenic response in TG2 knockout mice was reduced when compared to wild type animals of the same mouse strain (Figure 6C–D).

## Discussion

Previous *in vitro* findings suggest that celiac disease-specific TG2-targeted autoantibodies disturb angiogenesis in two-dimensional cell cultures [10], [14], [25]. The data presented in this article clearly demonstrate that celiac disease autoantibodies are anti-angiogenic also in more complex *ex vivo* systems using mouse aorta rings grown in three-dimensional conditions. Moreover, our data show for the first time that angiogenesis is hindered by celiac antibodies also *in vivo* in mice. In addition, the present results demonstrate that celiac disease-specific TG2 autoantibodies affect the functionality of vessels *in vivo*, which would confirm the previously suggested effects of celiac autoantibodies on endothelial permeability [11].

The data presented in Figure 3 strongly suggest that the anti-angiogenic effects of celiac antibodies are attributable to affected endothelial cell mobility, but also their reduced capacity to form sprouts and to establish cell-cell contacts. During the angiogenic process, the breakdown of the matrix in the leading edge of a migrating cell is a prerequisite for proper movement [26], [27]. In cultures supplemented with CD Mab, such areas of proteolytic degradation were not present and instead the cells were surrounded by electron-dense areas of ECM. These observations would indicate that in the presence of celiac autoantibodies ECM degradation/remodeling is disturbed, which could contribute to defective mobility discussed above. Interestingly, it has been shown that the administration of catalytically active TG2 causes an accumulation of ECM and defective angiogenesis *in vitro* [9]. Together this and our current finding, that cell-impermeable TG2-inhibitor R281 prevented the anti-angiogenic effects of celiac antibodies, would suggest that extracellular TG2 activity is involved in impaired angiogenic response in the presence of celiac antibodies. Another plausible explanation how R281 could rescue impaired angiogenesis is by preventing the binding of the celiac antibodies to TG2 but according to previous data [11] this did not seem to be the case.

Results obtained from experiments performed with TG2 knockout mice showed that in the *in vivo* matrigel plug *de novo* angiogenesis assay, where only the extracellular TG2 was present supplied by the matrigel itself, the angiogenic response was significantly decreased after CD Mab treatment. This observation underlines the importance of extracellular TG2 in the anti-angiogenic effect exerted by celiac disease antibodies. In the aorta ring experiments there was only a trend towards reduced microvessel and maximum outgrowth areas in the presence of CD Mab, the change was not however statistically significant. These somewhat discrepant results could be partly explained by the fact that in the TG2 knockout mice the overall angiogenic response in matrigel was greatly reduced compared to wild type animals while in the aorta ring experiment the reduction was not that extensive. This is probably due to differences in experimental settings, because in the matrigel plug assay the cells first have to migrate to plug to form vessels, whereas in the aorta ring assay cells can initiate the angiogenic process immediately. It is possible that also other factors, such as the proportion endogenous ECM secreted by the aortic tissue or individual migrating cells, might contribute to discrepancy.

The data derived from *in vivo* Hoechst injections and PET scans suggest that the vessels formed in matrigel in the presence of celiac autoantibodies are functional, as they transported the marker molecules injected into the circulation. However, the vascular area was smaller in the groups treated with CD Mab in both experiments. This would suggest that the functionality of the vessels was impaired either due to increased permeability or immaturity of the vessels. Because the number of vessels and their diameter were diminished in the presence of celiac autoantibodies, it is reasonable to assume that the latter alternative, the immaturity of the vessels, could explain our findings of impaired functionality. A similar state of immaturity, lacking a sufficient smooth muscle supportive layer, has previously been demonstrated in the small-intestinal mucosa of celiac disease patients [4].

Our present findings show that the celiac disease-specific autoantibodies interfere with angiogenesis *in vivo*. Thus, TG2-targeted autoantibody deposits around blood vessels in celiac patient small bowel mucosa [7] could be anti-angiogenic and lead to altered small bowel mucosal vasculature. This abnormal vascular network would no longer be able to provide mechanical support to the villi and thereby contribute to the development of villus atrophy. Interestingly, the association between morphological changes in intestinal mucosa and angiogenesis is supported by a recent paper, which describes that olmesartan, one of several angiotensin II receptor antagonists used for management of hypertension, was found to be associated with unexplained chronic diarrhea and small intestinal villus atrophy in patients taking the drug [28]. The same drug has been published to reduce angiogenesis in mice [28], [29].

The changes in the small-bowel mucosal vasculature in celiac disease might not only affect the mucosal architecture; they may also contribute to the pathogenesis in other ways. The celiac autoantibodies acting in concert with other factors including inflammatory mediators [30] could also potentiate the small-bowel mucosal inflammatory response by increasing the permeability of the vessels.

We conclude that celiac autoantibodies inhibit angiogenesis in three-dimensional *ex vivo* experiments, but also importantly *in vivo* in mice. The data presented in this article suggest that the anti-angiogenic mechanism of the disease-specific autoantibodies involves extracellular TG2 activity contributing to disruption of ECM remodeling. These could lead to inhibited endothelial cell mobility and finally to impaired angiogenesis. Defective angiogenesis could be coupled with compromised vascular function induced by celiac patient TG2-targeted autoantibodies and explain the findings of abnormal small-bowel vasculature in untreated celiac disease.

## Supporting Information

Figure S1 **Three-dimensional endothelial cell tubule formation assay.** Several angiogenic parameters quantified from human umbilical vein endothelial cells cultured inside matrigel for 48 h without any supplementation (basal) or in the presence of celiac patient-derived total IgA (CD IgA) or monoclonal antibodies (CD Mab), or their relevant controls (non-CD IgA or non-CD Mab; n = 9). Bars represent the average value as percentage + SEM. All data was normalized to the basal group (dotted line). * represents P≤0.001 statistical difference.
(TIF)

Figure S2 **The percentage of apoptotic cells in the presence of celiac antibodies** Apoptotic human umbilical vein endothelial cells inside matrigel cultures without any supplementation (basal) or in the presence of celiac patient-derived total IgA (CD IgA) or its respective control (non-CD IgA), or monoclonal celiac or control antibodies (CD Mab or non-CD Mab, respectively) were enumerated after 1, 15, 30 and 48 hours of culture with Cell-IQ from the videos. Results are given as percentages of total cell number.
(TIF)

Video S1 **Tubule dynamics of endothelial cells supplemented with celiac or control antibodies.** Human umbilical vein endothelial cells were grown inside matrigel in the presence of celiac patient-derived total IgA (CD IgA) or monoclonal antibodies (CD Mab), or their relevant controls (non-CD IgA or non-CD Mab) for ten days in a Cell-IQ system. During the assay pictures were taken every five minutes.
(MPEG)

Video S2 **Positron emission tomography (PET) and positron emission tomography/computed tomography (PET/CT) scanning 3D video from a mouse with matrigel implants.** PET and PET/CT scanning video of a mouse with matrigel implants without any supplementation (basal) or supplemented with celiac disease-specific transglutaminase 2-targeted monoclonal autoantibodies (CD Mab) or its relevant control (non-CD Mab). One mouse received three implants (basal, CD Mab or non-CD Mab), each injected subcutaneously into separate limbs as highlighted by circles in the video.
(MPEG)
