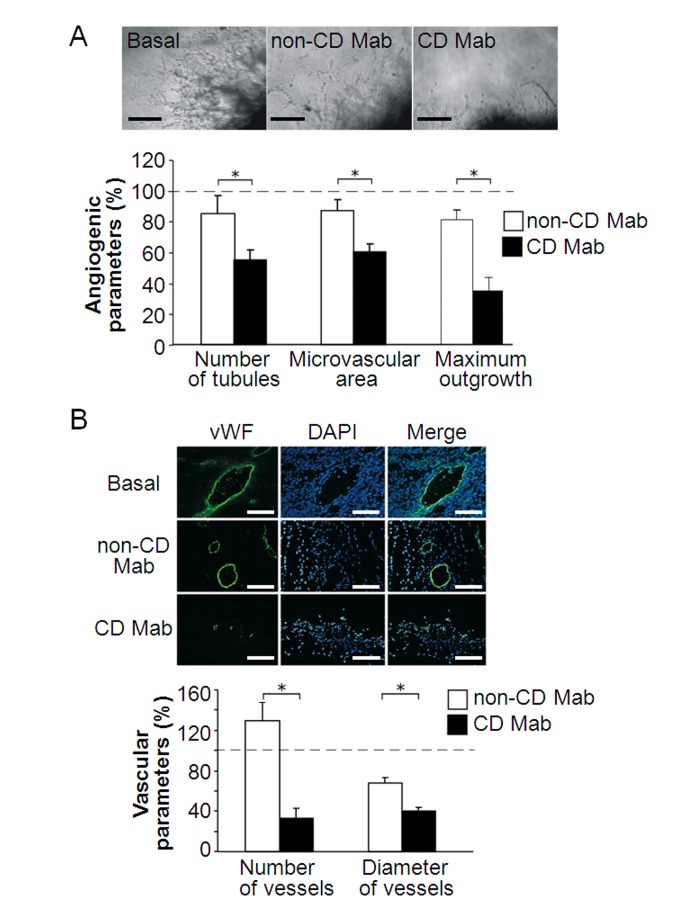# Correction: Celiac Disease–Specific TG2-Targeted Autoantibodies Inhibit Angiogenesis *Ex Vivo* and *In Vivo* in Mice by Interfering with Endothelial Cell Dynamics

**DOI:** 10.1371/annotation/a0040512-ea12-47b4-943b-2e68d665a851

**Published:** 2014-01-06

**Authors:** Suvi Kalliokoski, Ana-Marija Sulic, Ilma R. Korponay-Szabó, Zsuzsa Szondy, Rafael Frias, Mileidys Alea Perez, Stefania Martucciello, Anne Roivainen, Lauri J. Pelliniemi, Carla Esposito, Martin Griffin, Daniele Sblattero, Markku Mäki, Katri Kaukinen, Katri Lindfors, Sergio Caja

The labels "Basal" and "CD Mab" were erroneously switched in Figure 1B. Please see the corrected Figure 1 here: 

**Figure:**