# Supplementary figures and images for: Celiac Disease–Specific TG2-Targeted Autoantibodies Inhibit Angiogenesis Ex Vivo and In Vivo in Mice by Interfering with Endothelial Cell Dynamics
Source: PLoS One. 2013 Jun 18;8(6):e65887. doi: 10.1371/journal.pone.0065887 (PMC3688809; doi:10.1371/journal.pone.0065887)

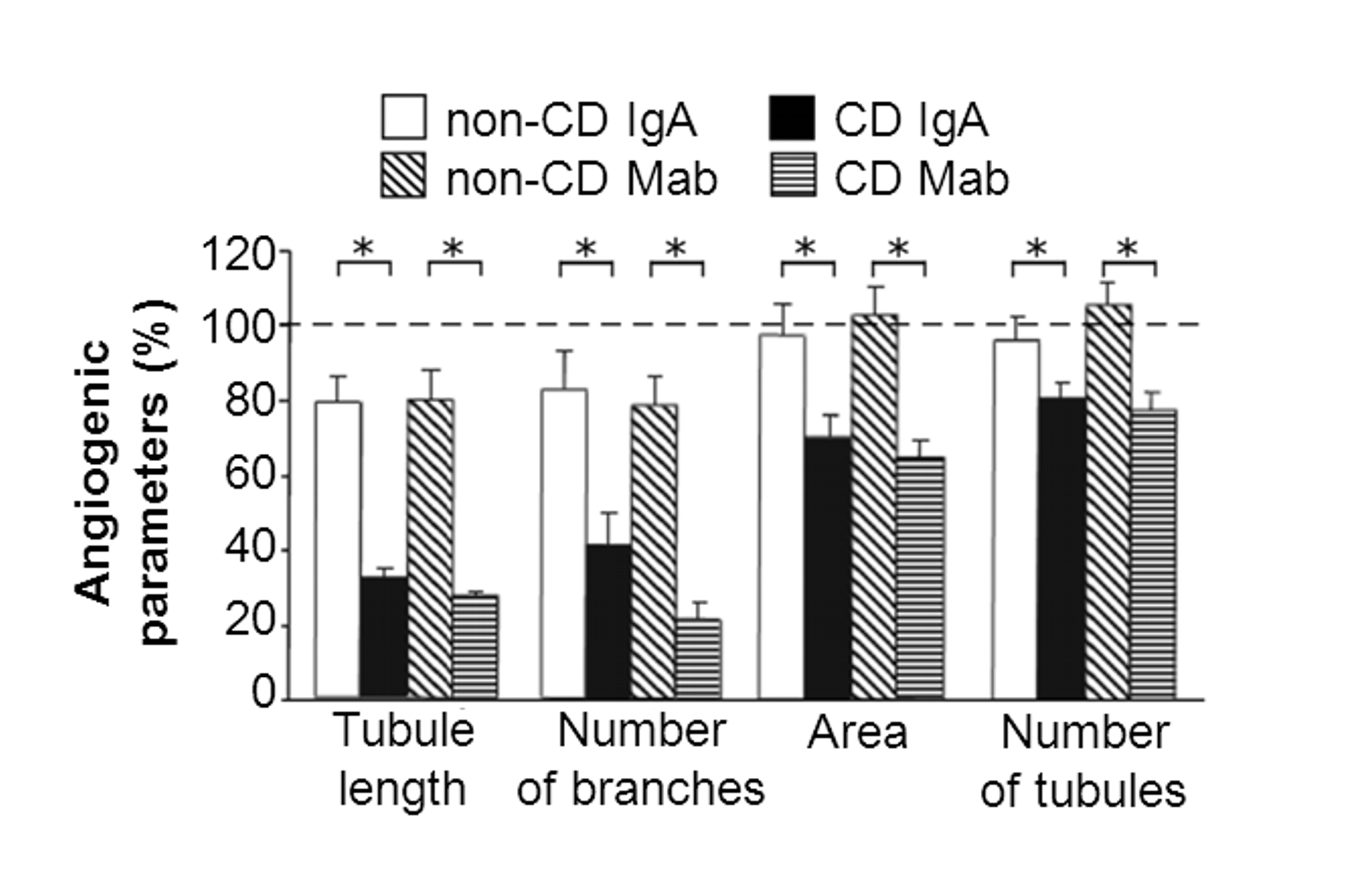

Supplement: Figure S1 — Three-dimensional endothelial cell tubule formation assay. Several angiogenic parameters quantified from human umbilical vein endothelial cells cultured inside matrigel for 48 h without any supplementation (basal) or in the presence of celiac patient-derived total IgA (CD IgA) or monoclonal antibodies (CD Mab), or their relevant controls (non-CD IgA or non-CD Mab; n = 9). Bars represent the average value as percentage + SEM. All data was normalized to the basal group (dotted line). * represents P≤0.001 statistical difference. (TIF) [file pone.0065887.s001.tif]

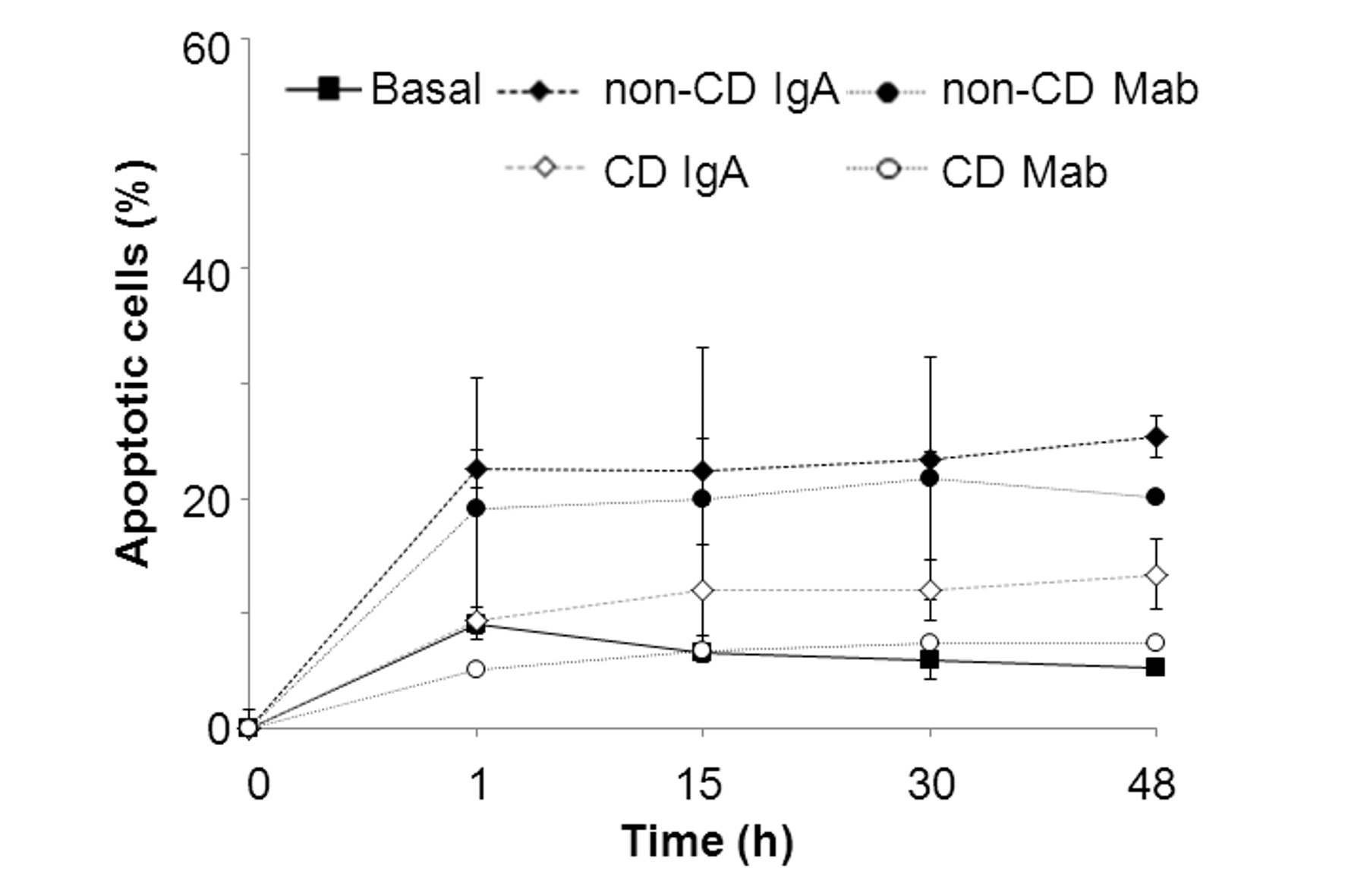

Supplement: Figure S2 — The percentage of apoptotic cells in the presence of celiac antibodies Apoptotic human umbilical vein endothelial cells inside matrigel cultures without any supplementation (basal) or in the presence of celiac patient-derived total IgA (CD IgA) or its respective control (non-CD IgA), or monoclonal celiac or control antibodies (CD Mab or non-CD Mab, respectively) were enumerated after 1, 15, 30 and 48 hours of culture with Cell-IQ from the videos. Results are given as percentages of total cell number. (TIF) [file pone.0065887.s002.tif]
